# Environmental noise perception and risk of poor mental health in a region on the Mediterranean coast of Spain

**DOI:** 10.1265/ehpm.25-00015

**Published:** 2025-05-10

**Authors:** Andreu Nolasco, Jesús Rabasco, Nayara Tamayo-Fonseca, Javier Casillas-Clot, Pamela Pereyra-Zamora

**Affiliations:** Research Unit for the Analysis of Mortality and Health Statistics. Community Health Research Group. Department of Community Nursing, Preventive Medicine, Public Health and History of Science, University of Alicante, Alicante, Spain

**Keywords:** Mental health, Noise, Environmental pollution, Spain, Health surveys, GHQ

## Abstract

**Background:**

Exposure to environmental noise may have a negative impact on a population’s mental health. We estimated the prevalence of exposure perception to high environmental noise in the Valencian Community, a region on the Mediterranean coast of Spain, and analysed its association with poor mental health risk, adjusting for demographic, socioeconomic and health status variables.

**Methods:**

Cross-sectional study based on a sample of 5.485 subjects, aged 15 or above, of the 2016 Valencian Community Health Survey. The risk of poor mental health was assessed via Goldberg’s questionnaire, a highly standardized self-reported questionnaire designed to screen for general psychological distress in the general population. Noise perception were determined in the home environment based on individuals’ responses to the Valencian Survey question about external noise problems. Sociodemographic variables, such as sex, age, level of education, or country of birth, and health variables, such as self-perceived health, or chronic diseases, were also considered. Logistic regression was used to estimate the Odds Ratios and confidence intervals of association between variables according to sex.

**Results:**

The prevalence of poor mental health was 26.2% [n = 2665; 95% CI: 24.5–27.9] in men and 33.6% [n = 2820; 95% CI: 31.9–35.3] in women. A total of 7.8% [n = 5485; 95% CI: 6.8–8.8] presented exposure to high noise perception, with no differences according to sex. Being at risk of poor mental health was significantly associated (p < 0.05) with high noise perception after adjusting for the rest of the variables (OR: 2.16 [95% CI: 1.46–3.19] in men; 2.46 [95% CI: 1.72–3.50] in women).

**Conclusions:**

Although the prevalence of exposure perception to high noise was not very high, population subgroups presenting high values were detected. High noise perception was related to the risk of poor mental health, regardless of other variables. Poor mental health risk was associated with exposure perception to high noise, other socioeconomic determinants, and health status. Improving noise exposure conditions could reduce the risk of poor mental health.

## Background

According to the WHO, exposure to environmental noise represents a significant risk to the population’s physical health, mental health, and well-being [1]. Data from the European Environment Agency show that 12,000 premature deaths occur as a result of such exposure each year, 6.5 million people suffer from sleep disturbances, and 22 million suffer from chronic discomfort in Europe [2]. In addition, the number of disability-adjusted life years in Western European countries is estimated to be 61,000 years for ischemic heart disease, 45,000 for cognitive impairment in children, 654,000 for discomfort, and 903,000 years for sleep disorders [1].

Noise, defined as “unwanted or harmful external sound”, is being increasingly perceived as an environmental disturbance and annoyance, and thus represents a public health problem [1, 3, 4]. Noise producers include road traffic, railways, airports, industrial sites, community, domestic and residential noise. In Europe, road traffic noise is the most dominant source of environmental noise, where at least 20% of population lives in areas with noise levels that are harmful to health [2].

Road traffic is the largest source of discomfort and is related to anxiety, depression and loss of well-being [1, 2, 5, 6]. Noise can cause individual adaptations. When it exceeds certain thresholds of duration and intensity – which differ according to each person – it can generate adverse health effects, such as stress, cardiovascular conditions, and lack of sleep [7].

Moreover, it has been observed that more than a third of the European population suffers from mental health disorders – the most common being depression and anxiety [8]. A 2014 study found that globally, one in five adults suffered a mental disorder over the last 12 months [9].

The relationship between noise exposure and the development of mental disorders has been demonstrated in several studies. Nevertheless, noise-related effects on mental health, as opposed to physical effects, have not been sufficiently addressed and results are often inconclusive [5, 10, 11]. In children, positive relationships have been found between exposure to traffic noise, airplanes and home noise and behavioural problems, hyperactivity, impaired memory and reading comprehension [12]. Cognitive and physical disorders, together with mental health problems, have been directly linked to acute noise exposure, which leads to physiological arousal through the stimulation of the endocrine system and the autonomic nervous system. The latter increases catecholamine and cortisol hormone levels, which could be the cause of health problems [13]. In addition, other variables such as sex, age, socioeconomic status, educational level, and chronic diseases are known to be potential predictors of this relationship [3, 14, 15].

In Spain, as in Europe, the main source of noise pollution by a significant margin, is road traffic [2]. It is estimated that in Spain, the percentage of inhabitants in urban areas exposed to unhealthy levels of road traffic noise is over 40%, and in large cities such as Valencia, the estimate of the population that may be affected by this source of noise reaches up to 68% [16, 17].

Regarding the studies conducted in Spain, most have sought to analyse how noise is associated with the effects produced on health in general – not exclusively on mental health. In addition, most of these studies have centred on the cities of Madrid and Barcelona, with the data of other regions remaining unknown. A number of these works address the following topics: the association of noise with mortality from cardiovascular, respiratory and metabolic diseases [18–21]; the effects of noise on the population in more disadvantaged neighbourhoods [22]; the association between long-term exposure to traffic noise and prevalent hypertension [23]; and the correlation between traffic noise and healthcare in Parkinson’s disease [24].

Previous works have raised the need to investigate the relationship between noise exposure and mental health through population-based studies. Given the lack of these types of studies in Spain, especially in regions other than those mentioned above, the objective of our work was to estimate the prevalence of exposure perception to high environmental noise in the Valencian Community. We also sought to analyse its association with the risk of poor mental health adjusting for demographic, socioeconomic and health status environment variables.

## Methods

### Design and sample

The design was cross-sectional and the population under study consisted of all non-institutionalised people aged 15 years or over, residents in the Valencian Community, a region on the Spanish Mediterranean coast. The data analysed correspond to the sample of 5,485 subjects – 2,665 (48.6%) men and 2,820 (51.4%) women – collected from the Health Interview Survey of the Valencian Community 2016 (HISVC-2016) [25].

The HISVC-2016 data collection was carried out between May and December 2016, on a sample of 5,280 dwellings, identified from the Population Information System of the Regional Ministry of Universal Health and Public Health. The sample subjects were selected using a complex sampling design that assigned each subject a weighting according to their representativeness. The weights were included in the HISVC-2016 databases provided by the Health Policy Planning and Evaluation Service of the Regional Ministry of Universal Health and Public Health of the Generalitat Valenciana, Spain.

The information was collected through a personal interview at the respondent’s home, assisted by tablets and other mobile devices. The interviewer completed the questionnaire, and the selected adult provided the answers.

### Variables

The main variable was exposure to the perception of environmental noise. This variable was based on the HISVC-2016 question: ‘Are there noise problems in your home coming from outside the house?’. Possible answers were: ‘A lot’, ‘A little’, and ‘None at all’. For the analyses, the answers were grouped under either ‘High noise perception’ (A lot) or ‘Low noise perception’ (A little or none at all).

The socioeconomic and demographic variables included: Sex (Male, Female), Age (15–44, 45–64, 65–84, and 85 years or older), Level of education (No studies, Primary, Secondary, University), Country of birth (Spain, Other country), Income perception (level of income self-perceived as Medium/Low, High), Employment status (Working, Retired, Unemployed, Other situations), Occupational class (Class I: Directors and managers of entities with 10 or more employees, and professionals traditionally associated with university degrees; Class II: Directors and managers of entities with less than 10 employees and professionals traditionally associated with higher education diplomas and other technical support professionals, athletes and artists; Class III: Intermediate occupations and self-employed workers; Class IV: Supervisors and workers in skilled technical occupations; Class V: Skilled workers in the primary sector and other semi-skilled workers; Class VI: Unskilled workers); Rural (Residence in municipality >10,000 or ≤10,000 inhabitants); Marital status (Single, Married, Separated-Divorced, Widowed), Cohabitating couple (Yes, No).

The health status variables analysed were self-perceived health (Poor, Good); severity index (or also known as sum score), based on the descriptive system of EuroQol-5D-5L health-related quality of life questionnaire (HRQoL), with values from 0 to 100 – 0 representing the total absence of health problems and 100 the worst possible health – and self-test thermometer of the state of health, based on the EuroQol Visual Analogue Scale with a score from 0 (worst state of health imaginable) to 100 (best state of health imaginable) [25, 26]. Chronic disease (Yes, No) and depression and/or anxiety (Yes, No) were included. These variables were obtained through the questions: “Has a doctor told you that you suffer from any of the following conditions...?” Participants who answered “yes” to any of the possible responses in the list of chronic diseases or to the questions about chronic anxiety and depression were included as “Yes” in the variables.

To analyse the association between noise perception and the risk of poor mental health, the result of the abbreviated, twelve-question version of Goldberg’s General Health Questionnaire (GHQ-12) was used as a response variable. The dichotomized score ranged from 0 to 12 points, from best to worst mental health: scores equal to or above 3 points corresponded to a ‘positive case, at risk of poor mental health’ (GHQ +) and the remaining scores were defined as a ‘negative case, no risk of poor mental health’ (GHQ −) [25, 27, 28].

### Data analysis

Contingency tables were elaborated to estimate and describe the prevalence of high noise perception (proportion as a percentage of people who reported high exposure to environmental noise) according to the different variable categories using Pearson’s Chi-square test to verify any significant differences (p < 0.05). For the estimated prevalence, 95% confidence intervals (95% CI) were constructed. For the severity index and the thermometer of health, means, standard deviations and 95% CI were calculated according to noise perception levels. Significant differences were established using Student’s t-tests.

The crude and adjusted associations between variables were estimated through Odds Ratios (OR) and 95% confidence intervals (95% CI) and were obtained from single and multiple binary logistic regression models. The GHQ result was considered as the response variable, and noise perception (main risk factor) and the rest of demographic, socioeconomic and health status variables (adjustment variables) were regarded as explanatory variables.

Due to the complex sample design of the survey, the weights provided in the survey were used to produce all the estimations. All analyses were distinguished by sex and performed using the SPSS^®^ software, version 25.

## Results

Regarding the first study objective, a prevalence of exposure to high environmental noise perception of 7.8% (n = 5,485) was found with no significant differences by sex (7.9% (n = 2,665) in men [6.9–8.9] and 7.7% (n = 2,820) in women [6.7–8.7]), according to the Valencian Community respondents.

Table 1 shows the prevalence and 95% CI according to categories of the explanatory variables and separated by sex. In the case of men, high noise perception profiles presented the following characteristics (higher percentage): middle-aged male (45–64 years), foreigner, separated marital status, unskilled worker, perception of medium or low income, self-perceived poor health, presence of chronic disease, with depression or anxiety, and residing in a municipality of over 10,000 inhabitants, with significant differences compared to other categories (p < 0.05). No significant differences were found according to educational level, employment situation, and cohabitating couple. In the case of women, high noise perception profiles presented the following features: middle-aged/elderly women, separated marital status, unskilled workers, low education level (no studies or primary level), unemployed, and with depression or anxiety.

**Table 1:** Frequencies, 95% CI, and observed prevalence of high noise according to explanatory variables by sex.

**Variable**	**Men**				**Women**			
	
	**n**	**PHNP^a^**	**95% CI**	***p***	**n**	**PHNP^a^**	**95% CI**	***p***
TOTAL	2665	7.9	[6.9–8.9]		2820	7.7	[6.7–8.7]	
AGE								
>85	62	4.8	[0–10.1]	0.006	116	6.9	[2.3–11.5]	0.035
65–84	467	7.3	[4.9–9.7]		563	8.7	[6.4–11.0]	
45–64	876	10.5	[8.5–12.5]		899	9.5	[7.6–11.4]	
15–44	1260	6.5	[5.1–7.9]		1240	6.3	[4.9–7.7]	
LEVEL OF EDUCATION								
No studies	257	7.0	[3.9–10.1]	0.612	346	8.4	[5.5–11.3]	0.001
Primary	724	7.0	[5.1–8.0]		731	10.9	[8.6–13.2]	
Secondary	1144	8.5	[6.9–10.1]		1081	6.7	[5.2–8.2]	
University	539	8.5	[6.1–10.9]		652	5.5	[3.8–7.2]	
COUNTRY OF BIRTH								
Other country	375	12.8	[9.4–16.2]	<0.001	360	7.5	[4.8–10.2]	0.853
Spain	2288	7.1	[6.0–8.2]		2455	7.8	[6.7–8.9]	
INCOME PERCEPTION								
Medium/Low	2244	8.2	[7.1–9.3]	0.03	2415	8.2	[7.1–9.3]	0.067
High	219	4.1	[1.5–6.7]		182	4.4	[1.4–7.4]	
EMPLOYMENT STATUS								
Other situations	408	10.3	[7.4–13.2]	0.146	730	9.5	[7.4–11.6]	0.008
Retired	608	8.7	[6.5–10.9]		544	7.2	[5.0–9.4]	
Unemployed	422	7.1	[4.6–9.6]		501	10.0	[7.4–12.6]	
Working	1223	7.0	[5.6–8.4]		1014	5.8	[4.4–7.2]	
OCCUPATIONAL CLASS								
Class VI	249	10.4	[6.6–14.2]	0.024	345	16.2	[12.3–20.1]	<0.001
Class V	868	8.2	[6.4–10.0]		748	7.9	[6.0–9.8]	
Class IV	457	8.5	[5.9–11.1]		453	7.3	[4.9–9.7]	
Class III	447	8.9	[6.3–11.5]		489	4.7	[2.8–6.6]	
Class II	299	3.0	[1.1–4.9]		328	4.6	[2.3–6.9]	
Class I	252	8.3	[4.9–11.7]		288	4.2	[1.9–6.5]	
MARITAL STATUS								
Separated	129	14.0	[8.0–20.0]	0.008	167	18.0	[12.2–23.8]	<0.001
Widow(er)s	88	5.7	[0.9–10.5]		355	6.2	[3.7–8.7]	
Married	1523	8.6	[7.2–10.0]		1441	7.5	[6.1–8.9]	
Single	924	6.2	[4.6–7.8]		850	6.7	[5.0–8.4]	
COHABITATING COUPLE								
No	1140	7.2	[5.7–8.7]	0.231	1388	7.3	[5.9–8.7]	0.357
Yes	1525	8.5	[7.1–9.9]		1426	8.2	[6.8–9.6]	
RURALITY								
>10,000 inhab.	2182	9.0	[7.8–10.2]	<0.001	2400	8.1	[7.0–9.2]	0.101
≤10,000 inhab.	482	2.9	[1.4–4.4]		417	5.8	[3.6–8]	
SELF-PERCEIVED HEALTH								
Poor	598	11.5	[8.9–14.1]	<0.001	886	7.9	[6.1–9.7]	0.828
Good	2067	6.9	[5.8–8.0]		1931	7.7	[6.5–8.9]	
CHRONIC DISEASE								
Yes	1224	10.2	[8.5–11.9]	<0.001	1492	8.0	[6.6–9.4]	0.625
No	1441	6.0	[4.8–7.2]		1323	7.5	[6.1–8.9]	
DEPRESSION/ANXIETY								
Yes	157	22.3	[15.8–28.8]	<0.001	274	12.4	[8.5–16.3]	0.002
No	2497	7.0	[6.0–8.0]		2540	7.2	[6.2–8.2]	

*Note.* CI = confidence interval;

^a^PHNP = Prevalence of high noise perception

When comparing men and women, a relationship was notably found between education level and employment status, with high noise perception in women but not in men. Moreover, a relationship with the variables of health status (self-perceived health and chronic disease), country of birth, income perception and rurality were found in men, but not in women.

Regarding the association between high noise perception and the severity index, a higher mean was found in both men and women compared to those who presented low noise perception. Regarding the association with the thermometer of health, the mean was slightly lower in people with high noise perception. (Table 2)

**Table 2:** Frequencies, mean, SD, 95% CI, according to noise perception by sex.

**VARIABLE**	**Men**					**Women**				
	
	**n**	**Mean**	**Standard deviation**	**95% CI**	***p***	**n**	**Mean**	**Standard deviation**	**95% CI**	***p***
SEVERITY INDEX^a^										
Low noise perception	2450	6.1	13.3	[5.6–6.7]	<0.001	2589	9.7	16.2	[9.0–10.3]	0.012
High noise perception	211	11.7	19.5	[9.0–14.3]		218	12.6	18.7	[10.1–15.1]	
THERMOMETER OF HEALTH^b^										
Low noise perception	2426	78.7	18.0	[78.0–79.4]	<0.001	2576	74.0	20.8	[73.2–74.8]	0.002
High noise perception	211	68.4	22.9	[65.3–71.5]		217	69.3	20.9	[66.5–72.1]	

*Note.* CI = confidence interval; SD = standard deviation; Scores based on the EuroQol.

^a^0 representing the total absence of health problems and 100 the worst possible health

^b^Score from 0 (worst state of health imaginable) to 100 (best state of health imaginable)

In terms of the relation with mental health, a prevalence of poor mental health of 30.0% was found: 26.2% in men and 33.6% in women. According to noise perception, these percentages were significantly different (p < 0.001). In the case of high noise perception, they reached values of 51.7% in men and 55.7% in women.

The male profile at risk of poor mental health included the following characteristics: exposure to high noise perception, advanced age, born in Spain, separate marital status, cohabitating couple, unskilled worker, and worker or supervisor in skilled technical occupation, presence of self-perceived poor health, presence of chronic disease, with depression or anxiety, and unemployed. In the case of women, the profile presenting the most frequent risk of poor mental health risk was as follows: exposure to high noise perception, advanced age, foreigner, widowed marital status, unskilled worker, no studies, self-perceived poor health, presence of chronic illness, with depression or anxiety, retired, and residing in a municipality of less than 10,000 inhabitants. When comparing men and women, it is worth highlighting that a significant relationship between education level and municipality size was found in the case of women but not men, while cohabitating couple was significant in men and not in women. Overall, the prevalence of risk of poor mental health was greater in women. (Table 3)

**Table 3:** Frequencies, 95% CI, and observed prevalence of poor mental health risk, according to explanatory variables.

**Variable**	**Men**				**Women**			
	
	**n**	**RPPMH^a^**	**95% CI**	***p***	**n**	**RPPMH^a^**	**95% CI**	***p***
TOTAL	2665	26.2	[24.5–27.9]		2820	33.6	[31.9–35.3]	
NOISE PERCEPTION								
High	211	51.7	[45.0–58.4]	<0.001	218	55.5	[48.9–62.1]	<0.001
Low	2453	24.0	[22.3–25.7]		2599	31.8	[30.0–33.6]	
AGE								
>85	62	41.9	[29.6–54.2]	<0.001	117	46.2	[37.2–55.2]	<0.001
65–84	467	25.9	[21.9–29.9]		564	38.7	[34.7–42.7]	
45–64	877	29.1	[26.1–32.1]		900	35.0	[31.9–38.1]	
15–44	1260	23.6	[21.3–25.9]		1240	29.2	[26.7–31.7]	
EDUCATION LEVEL								
No studies	258	31.0	[25.4–36.6]	0.069	346	43.9	[38.7–49.1]	<0.001
Primary	725	27.3	[24.1–30.5]		733	39.7	[36.2–43.2]	
Secondary	1144	26.0	[23.5–28.5]		1082	30.9	[28.1–33.7]	
University	539	22.6	[19.1–26.1]		654	26.3	[22.9–29.7]	
COUNTRY OF BIRTH								
Other country	376	21.8	[17.6–26.0]	0.04	361	42.4	[37.3–47.5]	<0.001
Spain	2288	26.8	[25.0–28.6]		2457	32.3	[30.5–34.1]	
INCOME PERCEPTION								
Medium/Low	2245	27.8	[25.9–29.7]	0.689	2418	35.2	[33.3–37.1]	0.056
High	219	26.5	[20.7–32.2]		181	28.2	[21.6–34.8]	
EMPLOYMENT STATUS								
Other situations	408	31.1	[26.6–35.6]	<0.001	730	36.2	[32.7–39.7]	<0.001
Retired	609	28.6	[25.0–32.2]		545	38.7	[34.6–42.8]	
Unemployed	421	33.3	[28.8–37.8]		501	35.1	[30.9–39.3]	
Working	1223	20.9	[18.6–23.2]		1015	28.0	[25.2–30.8]	
OCCUPATIONAL CLASS								
Class VI	249	30.9	[25.2–36.6]	<0.001	344	45.1	[39.8–50.4]	<0.001
Class V	869	25.5	[22.6–28.4]		748	29.1	[25.8–32.4]	
Class IV	457	30.9	[26.7–35.1]		453	40.0	[35.5–44.5]	
Class III	448	28.6	[24.4–32.8]		488	33.6	[29.4–37.8]	
Class II	298	14.4	[10.4–18.4]		327	27.2	[22.4–32.0]	
Class I	252	25.0	[19.7–30.3]		288	22.6	[17.8–27.4]	
MARITAL STATUS								
Separated	130	37.7	[29.4–46.0]	<0.001	168	35.7	[28.5–42.9]	<0.001
Widow(er)	88	34.1	[24.2–44.0]		356	46.3	[41.1–51.5]	
Married	1523	27.8	[25.5–30.1]		1443	31.9	[29.5–34.3]	
Single	924	21.1	[18.5–23.7]		851	31.0	[27.9–34.1]	
COHABITATING COUPLE								
No	1139	22.9	[20.5–25.3]	<0.001	1390	34.3	[31.8–36.8]	0.462
Yes	1526	28.6	[26.3–30.9]		1427	33.0	[30.6–35.4]	
MUNICIPALITY								
>10,000 inhab.	2183	25.5	[23.7–27.3]	0.11	2402	32.8	[30.9–34.7]	0.023
≤10,000 inhab.	482	29.0	[24.9–33.1]		418	38.5	[33.8–43.2]	
SELF-PERCEIVED HEALTH								
Poor	598	40.8	[36.9–44.7]	<0.001	888	48.1	[44.8–51.4]	<0.001
Good	2066	21.9	[20.1–23.7]		1932	27.0	[25.0–29.0]	
CHRONIC DISEASE								
Yes	1224	30.0	[27.4–32.6]	<0.001	1495	37.8	[35.3–40.3]	<0.001
No	1441	23.0	[20.8–25.2]		1324	28.9	[26.5–31.3]	
DEPRESSION/ANXIETY								
Yes	157	53.5	[45.7–61.3]	<0.001	275	59.3	[53.5–65.1]	<0.001
No	2498	24.5	[22.8–26.2]		2542	30.8	[29.0–32.6]	

*Note.* CI = confidence interval

^a^RPPMH: Risk Prevalence of Poor Mental Health

Regarding the association between the risk of poor mental health (GHQ +) and the severity index, the means were higher in the positive cases in both men and women, suggesting a worse HRQoL when at risk of poor mental health. The highest mean was notably found in women. Concerning the thermometer of health results, the mean was higher in men and women who were not at risk of poor mental health, which indicated better general health in this group. All differences were significant (p < 0.001). (Table 4)

**Table 4:** Frequencies, mean, SD and 95% CI, according to poor mental health risk by sex.

**VARIABLE**	**Men**					**Women**				
	
	**n**	**Mean**	**Standard deviation**	**95% CI**	***p***	**n**	**Mean**	**Standard deviation**	**95% CI**	***p***
SEVERITY INDEX^a^										
GHQ −	1966	4.8	11.4	[4.3–5.3]	<0.001	1863	6.7	12.4	[6.1–7.2]	<0.001
GHQ +	695	11.6	18.7	[10.2–13.0]		947	16.2	21.0	[14.9–17.6]	
THERMOMETER OF HEALTH^b^										
GHQ −	1945	80.5	16.4	[79.7–81.2]	<0.001	1857	77.7	18.6	[76.8–78.5]	<0.001
GHQ +	692	70.6	22.4	[68.9–72.3]		939	65.6	22.7	[64.1–67.0]	

*Note.* CI = confidence interval; SD = standard deviation

^a^0 representing the total absence of health problems and 100 the worst possible health

^b^Score from 0 (worst state of health imaginable) to 100 (best state of health imaginable)

Table 5 shows the OR (95% CI) of the association between the risk of poor mental health and the explanatory variables studied, distinguished by sex. In men, in the simple analysis, all variables except income perception and rurality were significantly associated (p < 0.05) with the risk of poor mental health. They were also all significantly associated in women, except the variables: cohabitating couple, rurality, and income perception. When the model was adjusted including all the explanatory variables, for men, the association was maintained in the case of those presenting high noise perception, born in Spain, being unemployed or in other situations, separated/divorced or widowed marital status, cohabitating couple, with poor self-perceived health, absence of chronic disease, and presence of depression or anxiety. It also showed a significant association with the severity index (higher risk of poor mental health with a higher severity index) and the thermometer of health (higher risk of poor mental health with poorer health).

**Table 5:** OR and 95% CI between poor mental health risk and variables studied, by sex.

**VARIABLE**	**Men**				**Women**			
	
	**Simple analysis**		**Multivariate analysis**		**Simple analysis**		**Multivariate analysis**	
			
	**OR**	**95% CI**	**OR**	**95% CI**	**OR**	**95% CI**	**OR**	**95% CI**
NOISE PERCEPTION								
High	**2.77**	[1.93–3.98]	**2.16**	[1.46–3.19]	**2.64**	[1.89–3.70]	**2.46**	[1.72–3.50]
Low	1		1		1		1	
AGE								
>85	**2.05**	[1.04–4.07]	0.58	[0.22–1.53]	**2.31**	[1.37–3.90]	0.49	[0.22–1.09]
65–84	1.12	[0.83–1.53]	**0.38**	[0.19–0.75]	**1.43**	[1.10–1.85]	0.72	[0.43–1.18]
45–64	1.26	[0.98–1.62]	**0.52**	[0.37–0.73]	1.22	[0.98–1.51]	1.04	[0.80–1.36]
15–44	1		1		1		1	
LEVEL OF EDUCATION								
No studies	**1.70**	[1.10–2.60]	0.87	[0.52–1.48]	**2.07**	[1.47–2.92]	0.98	[0.61–1.57]
Primary	**1.44**	[1.03–2.00]	0.97	[0.64–1.45]	**1.70**	[1.30–2.24]	1.35	[0.97–1.87]
Secondary	1.26	[0.92–1.71]	1.04	[0.74–1.48]	1.09	[0.85–1.42]	0.95	[0.72–1.25]
University	1		1		1		1	
COUNTRY OF BIRTH								
Other country	**0.63**	[0.45–0.90]	**0.58**	[0.39–0.85]	**1.72**	[1.31–2.25]	**2.00**	[1.51–2.64]
Spain	1		1		1		1	
INCOME PERCEPTION								
Medium/Low	1.08	[0.73–1.59]	0.70	[0.47–1.06]	1.37	[0.93–2.00]	0.99	[0.67–1.49]
High	1		1		1		1	
EMPLOYMENT STATUS								
Other situations	**1.93**	[1.39–2.69]	**3.19**	[2.08–4.87]	**1.40**	[1.10–1.79]	0.88	[0.67–1.16]
Retired	**1.53**	[1.16–2.02]	1.29	[0.71–2.31]	**1.44**	[1.10–1.88]	0.69	[0.43–1.12]
Unemployed	**1.68**	[1.24–2.29]	**2.49**	[1.77–3.52]	**1.42**	[1.08–1.86]	1.13	[0.85–1.51]
Working	1		1		1		1	
OCCUPATIONAL CLASS								
VI	1.22	[0.76–1.98]	0.88	[0.50–1.53]	**2.94**	[1.94–4.44]	**1.65**	[1.05–2.60]
V	0.99	[0.66–1.47]	1.03	[0.65–1.65]	1.33	[0.91–1.95]	0.89	[0.59–1.34]
IV	1.28	[0.83–1.97]	1.33	[0.81–2.18]	**2.41**	[1.62–3.60]	**1.89**	[1.23–2.88]
III	1.00	[0.64–1.54]	1.18	[0.74–1.90]	**1.70**	[1.14–2.54]	1.46	[0.96–2.20]
II	**0.51**	[0.30–0.87]	0.64	[0.37–1.10]	1.28	[0.83–1.97]	1.20	[0.78–1.86]
I	1		1		1		1	
MARITAL STATUS								
Separated/Divorced	**2.38**	[1.44–3.92]	**3.04**	[1.70–5.43]	1.23	[0.81–1.85]	0.64	[0.39–1.03]
Widow(er)	1.70	[0.92–3.15]	**2.63**	[1.24–5.57]	**2.18**	[1.57–3.02]	1.21	[0.77–1.91]
Married	**1.36**	[1.06–1.73]	1.28	[0.79–2.07]	1.02	[0.82–1.27]	**0.54**	[0.35–0.82]
Single	1		1		1		1	
COHABITATING COUPLE								
No	**0.77**	[0.61–0.96]	**0.46**	[0.29–0.73]	1.09	[0.90–1.32]	0.72	[0.48–1.07]
Yes	1		1		1		1	
RURALITY								
>10,000 inhab.	0.81	[0.62–1.07]	**0.71**	[0.53–0.95]	0.87	[0.67–1.13]	0.87	[0.66–1.14]
≤10,000 inhab.	1		1		1		1	
SELF-PERCEIVED HEALTH								
Poor	**2.62**	[2.06–3.35]	**1.49**	[1.03–2.15]	**2.56**	[2.10–3.12]	1.02	[0.75–1.38]
Good	1		1		1		1	
CHRONIC DISEASE								
Yes	**1.37**	[1.10–1.71]	**0.69**	[0.52–0.90]	**1.49**	[1.23–1.81]	**0.69**	[0.54–0.88]
No	1		1		1		1	
DEPRESSION/ANXIETY								
Yes	**3.63**	[2.39–5.51]	**2.13**	[1.28–3.52]	**3.35**	[2.44–4.60]	**1.75**	[1.19–2.55]
No	1		1		1		1	
SEVERITY INDEX^a^	**1.03**	[1.03–1.04]	**1.01**	[1.00–1.02]	**1.03**	[1.03–1.05]	**1.03**	[1.02–1.04]
THERMOMETER OF HEALTH^a^	**0.97**	[0.97–0.98]	**0.98**	[0.97–0.99]	**0.97**	[0.97–0.98]	**0.98**	[0.97–0.98]

*Note.* CI = confidence interval; Bold indicates statistically significant values (p < 0.05). The multivariate model in both men and women was adjusted for all explanatory variables. ^a^OR represents the change in the odds of the outcome for each unit increase in the variable.

In the case of women, the poor mental health risk showed a significant adjusted association with high noise perception, being a foreigner, being an unskilled worker, being a worker or supervisor in a skilled technical profession and having depression or anxiety. There was a confounding effect (losing the statistical significance of the effect) on chronic disease, and marital status. A significant association was also found with the severity index and the thermometer of health scores, as in the case of men.

Of note, regarding the relation with noise, a significant association was maintained in both men and women and subjects with high noise perception presented a higher risk after adjusting for the rest of the variables.

## Discussion

The present study showed that the prevalence of exposure to high noise perception in the Valencian Community population was 7.8%, lower than in other European countries [2], or for Spain’s total population, where prevalences between 9.3% and 36.5% have been reported in the literature [15, 29, 30] Noise exposure presented a significant association with the risk of poor mental health. After adjusting the effect of noise on poor mental health risk to demographic, socioeconomic and health status explanatory variables, high noise perception remained significant in both sexes, and significant associations between poor mental health risk and other variables were obtained.

The risk of poor mental health affects up to 30.0% of the population, and was higher in women (33.6%), than in men (26.2%). These percentages were higher than those reported in the literature for the overall Spain and other regions, where the prevalence of psychological distress ranges between 19% and 22% had been reported [15, 31]. Similarly, higher percentages of distress for women are described for Spain and most regions. However, prevalences in most regions are lower, although with small exceptions, such as Andalusia or Murcia, where prevalences in women reached 34.2% and 35.3% in 2017 [31].

Also, our study showed that the prevalence of risk of poor mental health or psychological distress was higher among those who presented high noise perception and higher in women (reaching up to 51.7% in men and 55.7% in women). The trend is similar for Spain [15, 29], where higher prevalences of risk of poor mental health were found among those who had a high perception of noise and higher among women, but with lower prevalences than those found in our results, both for the population under 65 years of age (21.8% in men and 32.6% in women) and for the general population (17.4% in men and 29.5% in women).

The prevalence results, both for noise exposure and for the risk of poor mental health, presented a general socioeconomic gradient. The greatest exposure to noise in the lowest socioeconomic strata was usually related to the place of residence in cities [32, 33] and to greater exposure to aircraft or traffic noise [34]. People born in another country may present greater noise perception for these same reasons if they settle in areas with high noise exposure [32]. On the other hand, in line with other study, men with higher education levels were found to present a higher prevalence of noise exposure [35]. Contradictions can be found in the literature, however, owing perhaps to the different interpretations of high levels of education in countries and urban or rural areas [34].

The results showed that high noise perception was significantly associated with the population’s risk of poor mental health, regardless of other socioeconomic or geographical variables and health variables. These results coincide with those obtained in other studies [3, 14, 15, 36]. Studies that have used GHQ to assess mental health have linked aircraft noise and outside noise with the risk of poor mental health, especially in women [29, 37]. These results could be explained, as suggested in other studies, by the fact that people who indicated a high subjective noise perception were susceptible to it, noise becoming an annoyance and thus a stressor that negatively modified their mental health [11, 14, 37, 38]. In addition, mental health problems may increase noise perception, noise annoyance or noise sensitivity [11, 38–40], so further studies are necessary to clarify the problem of reverse causality and the association among those constructs.

In any event, the results seem to indicate that high noise perception is related to the risk of poor mental health regardless of whether the person is suffering from anxiety, depression or being in poor health. Given the lack of evidence on the subject, future studies should focus on analysing the effect of high environmental noise levels on different types of mental disorders. It will also be necessary in the future to conduct new intervention studies on the effect of modified environmental noise conditions, which may lead to improving the population’s quality of life.

### Limitations and strengths

The current study presented some limitations – notably the cross-sectional study design, which did not allow for establishing causal relationships. In the same way, it was not possible to achieve more accurate results for several reasons to be overcome in further studies: first, due to the unspecificity of the variable used to assess noise exposure, we could distinguish only between ‘High perception’ and ‘Low perception’.

Second, due to the lack of complementarity of subjective and self-reported measures to assess noise exposure, a triangulation of information with more objective and direct measures could not be performed. Despite this, the literature highlights the importance of considering perceived noise pollution (through the individuals’ subjective perception) given the substantial relationship between perceived noise and health problems [41]. Also, some studies have described a correlation between subjective perception variables and more objective measures of noise measurement [33, 42], which might suggest that individuals may be able to assess their own degree of exposure to risks. In this sense, it could be considered that a subjective measure of self-perception of environmental noise could be used, where appropriate, as a proxy measure of more objective measures. Regarding the measure used in this study, some European surveys have adopted slightly different formulations [43, 44], which should be taken into account when comparing across countries.

Third, owing to the absence of variables directly related to noise annoyance assessment or noise sensitivity, which has been described in the literature as a possible mediator between noise exposure and different health outcomes, including mental health in the population [11]. Finally, the lack of identification of the specific source of external noise (industry, nightlife areas, medium/high capacity roads, railways, airports, neighbourhood, etc.) as it has been done in another region of Spain [6], or the lack of information about exposure outside the home (during work, leisure, etc.). Future studies should consider household characteristics, such as household size and composition or family structure, (e.g., number of members, number of children under 15, older adults in multigenerational family households, children in single-parent households, single-person households, etc.) as potentially influential variables in mental health [45, 46]. Despite this, the results obtained are nevertheless highly consistent with the findings of other studies and can serve to guide future lines of research.

A major strength of this study was the use of a large sample size and data from a representative survey of the general population in the Valencian Community. The instrument employed to measure the risk of poor mental health, the GHQ-12, has been validated and is widely used for this purpose. This makes the results of this study highly replicable and generalizable to other populations with similar characteristics.

## Conclusions

The present work is one of the first studies to analyse the prevalence and impact of exposure perception to high noise in the Valencian Community, a region on the Mediterranean coast of Spain. The most disadvantaged socioeconomic strata presented greater noise perception, suggesting that poor quality of housing and the environment, among others, act as determinants. Poor mental health risk was higher in women and was related to exposure to high noise perception and other socioeconomic determinants and health status. Noise exposure may be considered as a mediating factor between such determinants and the risk of poor mental health. Improving noise exposure conditions (acoustic conditioning, traffic reduction, noise barriers, acoustically respectful standards of social behaviour, etc.) could lower the risk of poor mental health.

## Abbreviations

GHQ-12: Goldberg’s General Health Questionnaire
HISVC-2016: Health Interview Survey of the Valencian Community 2016
HRQoL: EuroQol-5D-5L health-related quality of life questionnaire
OR: Odds Ratios
PHNP: Prevalence of high noise perception
RPPMH: Risk Prevalence of Poor Mental Health
SD: Standard Deviation

## Declarations

### Availability of data and material

The data of the Health Interview Survey of the Valencian Community 2016 analysed for this study can be obtained from the Office of Health Plan (Conselleria de Sanitat of the Valencian Autonomous Government) on demand: https://www.san.gva.es/es/web/conselleria-de-sanidad-universal-y-salud-publica.
